# Attracting and retaining health workers in rural areas: investigating nurses’ views on rural posts and policy interventions

**DOI:** 10.1186/1472-6963-10-S1-S1

**Published:** 2010-07-02

**Authors:** Kethi Mullei, Sandra Mudhune, Jackline Wafula, Eunice Masamo, Michael English, Catherine Goodman, Mylene Lagarde, Duane Blaauw

**Affiliations:** 1 Monitoring and Evaluation – Research Management & Documentation Officer, IntraHealth International, Inc. P.O Box 66726, 00800, Nairobi, Kenya; 2 KEMRI-Wellcome Trust Research Programme, P.O Box 43460, Nairobi, Kenya; 3 District Health Management Team, Taveta District, Ministry of Public Health, Kenya; 4 Department of Pediatrics, University of Oxford, John Radcliffe Hospital, Oxford OX9 9DU, UK; 5 Health Economics and Financing Programme, London School of Hygiene & Tropical Medicine, Keppel St. London WC1E 7HT, UK; 6 Centre for Health Policy, School of Public Health, University of Witwatersrand, Johannesburg, South Africa

## Abstract

**Background:**

Kenya has bold plans for scaling up priority interventions nationwide, but faces major human resource challenges, with a lack of skilled workers especially in the most disadvantaged rural areas.

**Methods:**

We investigated reasons for poor recruitment and retention in rural areas and potential policy interventions through quantitative and qualitative data collection with nursing trainees. We interviewed 345 trainees from four purposively selected Medical Training Colleges (MTCs) (166 pre-service and 179 upgrading trainees with prior work experience). Each interviewee completed a self-administered questionnaire including likert scale responses to statements about rural areas and interventions, and focus group discussions (FGDs) were conducted at each MTC.

**Results:**

Likert scale responses indicated mixed perceptions of both living and working in rural areas, with a range of positive, negative and indifferent views expressed on average across different statements. The analysis showed that attitudes to working in rural areas were significantly positively affected by being older, but negatively affected by being an upgrading student. Attitudes to living in rural areas were significantly positively affected by being a student at the MTC furthest from Nairobi.

During FGDs trainees raised both positive and negative aspects of rural life. Positive aspects included lower costs of living and more autonomy at work. Negative issues included poor infrastructure, inadequate education facilities and opportunities, higher workloads, and inadequate supplies and supervision. Particular concern was expressed about working in communities dominated by other tribes, reflecting Kenya’s recent election-related violence.

Quantitative and qualitative data indicated that students believed several strategies could improve rural recruitment and retention, with particular emphasis on substantial rural allowances and the ability to choose their rural location. Other interventions highlighted included provision of decent housing, and more rapid career advancement. However, recently introduced short term contracts in named locations were not favoured due to their lack of pension plans and job security.

**Conclusions:**

This study identified a range of potential interventions to increase rural recruitment and retention, with those most favored by nursing students being additional rural allowances, and allowing choice of rural location. Greater investment is needed in information systems to evaluate the impact of such policies.

## Background

To achieve the United Nations Millennium Development Goals (MDGs) for health, improving access to key interventions such as anti-retroviral therapy, immunizations, and tuberculosis and malaria treatment, are top priorities for most health systems [1-3]. However, in addition to financial resources for commodities [4] scaling up requires a well-functioning health system and adequate workforce capable of delivering interventions at a large scale [5-9]. However, the 2004 Joint Learning Initiative report on Human Resources for Health and others have concluded that shortage and mal-distribution of health personnel, with few in rural areas, undermine scaling up efforts, particularly in low-income countries [10-13]. In sub-Saharan Africa it has been argued that nearly 1 million additional health workers are needed to realize MDG health outcomes [14].

In Kenya understaffing in rural primary care facilities (dispensaries and health centers) is particularly acute. Staffing norms for dispensaries are 2 to 5 nurses, but 9% have no nurse at all and 48% only one. Similarly, health centers should have 8 to 12 nurses, with the larger health centers expected to offer short-term residential basic obstetric care, but 48% have less than three [15] (physicians are not an expected part of the workforce at either of these rural facility types). The most obvious solution to address the rural and general nursing workforce shortage is a major increase in recruitment, as there are many qualified and unemployed nurses in Kenya [1]. In an attempt to address staff shortages quickly, donor partners have supported recruitment of just over 3000 nurses from 2005 to date, hired on short-term contracts of 2 to 3 years duration, and posted to public sector facilities in underserved rural areas with high HIV prevalence [16]. However, major shortages remain.

Posts in rural areas are often considered less desirable. Urban settings have superior infrastructure and services [3,17,18] while rural posts are often associated with high workloads and poorly aligned incentive systems (for example lower housing allowances) that disadvantage rural staff. Once they are posted in rural areas, some health workers make repeated demands to be reallocated to urban postings (Chief Nursing Officer, personal communication).

There are two main cadres of nurses in Kenya: registered nurses (RNs) and enrolled community nurses (ECNs). RNs and ECNs undergo a 3.5 year and 2.5 year pre-service training respectively. The majority of Kenya’s nursing colleges now train predominantly RNs (90% of trainees) while previously the majority of trainees were ECNs. Rural posts in health centers and dispensaries are currently mainly filled by ECNs while posts in hospitals are mainly filled by RNs. As of 2008, entry-level RNs and ECNs received monthly basic salaries of KES 22 519 (USD 289) and KES 11 518 (USD 148) respectively converted at a rate of (1 USD = KES 78.19) [19]. ECNs have the option to upgrade to RN status either through a 1.5 year on-campus program or a 2 year distance learning program, which many are currently choosing to do, given the salary differentials and the limited career progression for ECNs. A minority of RNs have a bachelor’s degree but this group is not considered in this paper.

The debate in Kenya over how to ensure adequate numbers of nurses in rural areas to provide and supervise essential community and primary care services is poorly informed by evidence. This study, focusing on RN trainees near graduation, set out to explore the views of final year nursing students on rural and urban areas, nursing posts in rural areas, and strategies to recruit and retain nurses. Results are presented separately for those completing pre-service training and those upgrading from ECN status, as variation in characteristics and experience between the two groups is expected to affect their attitudes.

## Methods

Between September 2008 and January 2009 data were collected from 345 RN students in their final year of training at four Medical Training Colleges (MTCs), using a structured self-administered questionnaire (SAQ), and focus group discussions (FGDs). MTCs were eligible for the study if they had at least 30 pre-service and 30 upgrading students scheduled to complete training in 2008. Of a total of eight eligible MTCs, four were purposively selected (Nairobi, Murang’a, Meru and Kakamega). Nairobi MTC is located in the capital city, while Murang’a, Meru and Kakamega are 87 km, 238 km and 355 km from Nairobi respectively, representing a range of settings across four provinces. All students who intended to sit their final exams in the second half of 2008 were invited to participate.

The SAQ data covered respondents’ backgrounds, training plus sets of likert scale statements, designed to assess their attitudes towards living and working in rural areas. The terms “rural” and “urban” are open to varied interpretation [20]. We advised students completing the SAQ to consider rural areas as those in remote places, far from major cities and towns and with poor infrastructure and limited recreational facilities, though they may also have incorporated their own individual views of these terms. Their interpretations were explored further in the FGDs. The development of the likert scale statements drew on those reported in the literature [21], adapted to improve applicability to the local context through discussions with locally qualified nursing staff and researchers. Statements were adapted to ensure that they covered all key issues raised by local stakeholders, that all issues were relevant to the Kenyan nursing profession, and that the language was easily understandable in the local context. Students were asked to respond to each statement on a 6 point scale of 1 (strongly disagree), 2 (moderately disagree), 3 (somewhat disagree), 4 (somewhat agree), 5 (moderately agree) and 6 (strongly agree). To reduce response bias, 18% of the statements were phrased negatively, and randomly distributed within question sets. The SAQ tool was pre-tested with students from two MTCs not included in the study, including a group discussion of the meanings of the questions and statements.

Two FGDs were carried out in each MTC, one each for pre-service and upgrading students. 6-8 participants were randomly selected for each group from all those completing the SAQ. Each group included both male and female students. The discussions covered trainees’ experiences, attitudes towards rural areas, rural postings, and nursing in general, and potential interventions to improve health worker distribution. Discussions lasted between 45 and 90 minutes, and were digitally recorded, supplemented by note taking. Draft topic guides were piloted with nursing students from urban and rural MTCs.

The voluntary nature of participation was emphasized, and informed written consent was obtained from all participants. No MTC tutors were present during data collection.

Data from the SAQ were double-entered and verified. Simple descriptive analysis was used to compare the characteristics of pre-service and upgrading students, and to calculate the mean response (and 95% confidence interval) for each likert scale statement. As each likert scale statement was rated on a scale from 1 to 6 we considered an upper confidence interval less than 3.5 as an indication of general disagreement with the statement, and a lower confidence interval above 3.5 as an indication of general agreement. Principal components analysis (PCA), a data reduction technique that enables one to identify from a large set of variables those that contain information common to all, was performed across all statements on rural/urban perceptions to determine how the statements grouped together under the broad constructs of ‘perception of life in a rural area’ and ‘perception of working in rural areas’. Additional PCAs were then run within these two constructs, the first component for each construct was retained and PCA derived scores calculated for each construct. These scores were used as dependant variables in separate linear regressions to assess the influence of a range of trainee characteristics on attitudes to rural life and work. All analyses were conducted in STATA version 10.1 (Stata, http://www.stata.com).

FGD recordings and field notes were reviewed for clarity, transcribed, uploaded into the qualitative analysis software, *NVivo7*, and subjected to content analysis. This involved development of a coding “tree” or thematic framework. A draft coding tree was developed from the FGD topic guide, tested by two researchers separately on about 30% of all data collected, and refined using themes emerging from transcripts. Information under each code was then compiled and tabulated to obtain a clearer picture of the issues arising from the data, and to compare views across different groups of participants.

## Results

Results are presented on respondent characteristics, their perceptions of life and posts in rural areas, and their perceptions of strategies to improve rural recruitment and retention, drawing from both the SAQ and FGD data.

### Characteristics of respondents

Of the 462 students invited, 345 agreed to participate, giving a response rate of 92% for pre-service students and 63% for upgrading students. The lower rate amongst the latter was attributed to difficulties in communicating the invitations, long travel distances from work stations to data collection points, and students’ work commitments. Respondents were divided approximately equally between pre-service and upgrading students (48.1% and 51.9% respectively), and 34.5% were from the most urban MTC in Nairobi (Additional file 1). Two thirds described themselves as having been born in a rural or relatively rural area.

Most students were female (75.4%). The upgraders formed an older group, with a mean age of 38 years (range 24-52) compared with 24 years (range 20-40) for pre-service students. Upgraders were also more likely to be married and to have children (Table 1). Level of education of parents was higher for pre-service students than for upgraders (77.7% and 72.3% of pre-service students’ fathers and mothers, respectively, had completed primary education, compared to 57.5% and 40.2% for upgraders). For most students, parents and family members paid for their studies, with only a small proportion (9.9%) obtaining scholarships for their training, of which the majority (7%) received government scholarships. All pre-service interviewees were full-time on-campus students, while all upgrading interviewees were studying on a distance learning basis, reporting to the college for a few weeks to attend introduction to modules or to sit exams.

More upgrading trainees had chosen nursing as their first career choice (77.7%) compared to pre-service trainees (61.5%). In FGDs, some pre-service students stated that they had agreed to pursue nursing in response to parental pressure, or that nursing only became an option because they failed to get accepted into their preferred training programmes such as clinical medicine and pharmacy.

“…actually I applied for pharmacy…. but they took me for nursing and I had no choice because I had already applied three times, I was not going to lose the chance” Pre-service nursing student

Experience of working in rural areas was very different between the two groups. 68.7% of upgrading trainees had already held posts in rural areas, in contrast to pre-service students who had no rural (or urban) job experience.

When asked in FGDs which sector they would target upon graduation, upgrading students did not appear to have a strong preference for any particular sector but a minority of pre-service students favored private hospitals and non-governmental organizations (NGOs) which are mainly located in urban areas. These facilities were associated with higher salaries.

“I would prefer after college to go maybe to private sector because there (for) one, I’m assured of a good salary…” Pre-service nursing student

### Perceptions of rural areas

A preliminary PCA was conducted of statements numbered 1-12 in Additional File 2, which indicated that they could be broadly grouped into two domains. The first group of variables included statements 1-4 (Cronbach’s alpha of 0.66) which all described life in rural areas. The second construct pooled together statements 5-11 (Cronbach’s alpha of 0.51) which pertained to work in rural areas. One statement (number 12) did not fit within either domain.

#### Life in rural areas

Students’ responses in FGDs suggested they generally understood rural areas to be those which are fairly remote with poor infrastructure (bad roads, limited transport services, no electricity, poor mobile phone network, and low water supply), poor health services, limited variety of available housing and hardly any recreational facilities.

“…you know our country how it is, infrastructure is bad, if you take me to a remote area, there is no accessibility, there is no infrastructure, there are no telephones, and there are no roads, it rains…I stay there for months without talking to my people in the urban…” Upgrading nursing student

Comparatively, urban areas were perceived to be more accessible with stronger infrastructure, better health services and educational institutions, and a variety of recreational facilities. Upgrading students, who were mostly married, commented that they often get separated from their children when they took rural posts, as their children were schooled away from them in urban areas as rural schools were considered to be of substandard quality.

However, during FGDs, rural areas were more positively often associated with a lower cost of living, with lower housing rents, school fees, and food prices reported.

“…the nurses who are out there, me I don’t hear them complain, they can afford. They eat food from the shambas (farms); things are not as expensive…” Pre-service nursing student

“Like in Nairobi to get a one bed-roomed house you need around KES 8000 (USD 102.5) but like where I went for my district experience, you could get a house for KES 1500 (USD 19.2) per month” Pre-service nursing student

When asked whether they were willing to work in any rural area in the country, both groups of students expressed fear of working in communities dominated by other tribes. They attributed their fear to post election violence that cut across the country in late 2007 and early 2008, mainly affecting rural areas.

“…I was on my marks just in case something happens I run away, and in fact one of the facilities that I personally initiated, I never went there for a long time because of fear of the post election violence, so but once you know that there is that security you feel good” Upgrading nursing student

Students cited examples of colleagues and family members who were forcefully evicted from their homes or working areas because of ethnic differences.

“Like myself if am told I go to Mount Elgon that place called Ekopsiro, I would not go for one there are two colleagues whose lives went, so you could not admire going to that particular place” Upgrading nursing student

Likert scale responses contrasting life in rural and urban areas did not elicit strong views from pre-service or upgrading students (Table 2), though respondents gave some indication that they found rural housing and lifestyles unappealing. The PCA conducted for this construct (based on statements 1, 2, 3 & 4), produced a first principal component that accounted for 50.3% of the total variance, which was used to produce a score reporting individuals’ perceptions of life in rural areas (a higher PCA score indicated stronger preference for rural areas) (Additional file 3). We modeled the determinants of this index using multivariable regression with the following independent variables: trainee type (upgrading or pre-service), age, sex, marital status, having children, being born in a rural area, and location of MTC (Additional file 4). The only significant finding was that students attending Kakamega MTC had generally more positive perceptions of “life in rural areas”.

#### Nursing posts in rural areas

Likert scale responses indicated that nursing students (both pre-service and upgraders) had mixed perceptions of nursing posts in rural areas. On the one hand they associated them with lower incomes, slow career advancement and workplace stress, but on the other hand they did not feel that they would be frightened to work there, that it would be difficult to bring up children or that they would be without support from colleagues and supervisors (Table 2). PCA on the 7 variables in this domain (statements 5-11 in Table 2) produced a first component which accounted for 26% of the total variance (Table 3). From the regression, being an upgrading student had a significantly negative effect on preferences for working in rural areas, but being older had a positive effect (Table 4).

During discussions upgrading students shared the view that poor communication channels in rural areas limited the flow of information on training opportunities such as workshops and seminars. In addition, they revealed that staff shortages denied them the opportunity to pursue their studies because a replacement was not always available.

“…when you stay in rural community as a health worker, you might end up missing some of the privileges that people in town do enjoy like maybe there are some seminars and you are not aware…like someone working here at PGH [Provincial General Hospital] you may be coming to further your studies here at MTC unlike when you are in rural area, you just stagnate there, there is no advancement” Pre-service nursing student

When discussing provision of allowances for nursing posts, participants acknowledged the fact that rural posts attract lower housing allowances compared to those offered in urban posts. Upgrading students were however quick to point out that the higher allowances paid in urban posts were used to cater for higher living expenses in these areas.

Upgrading students held the view that positions in rural dispensaries or health centers allowed for more autonomy at work, reflecting the low number of staff per facility, and better coordination of activities. They alluded to the fact that division of work and assignment of duties is much easier with fewer staff.

“The work is there but, because in the rural we have three people, we know now it is the end of the month, we know we have ten reports to be written …. We know it is our responsibility so we know how to do division of work” Upgrading nursing student

However, some pre-service students held more negative opinions of working in rural facilities, stating that unfavorable work conditions, such as higher workloads, poor staffing, infrequent support supervision and inadequate equipment and supplies, would make it difficult to perform daily duties or limit them to managing minor cases.

“You know when you are working in the rural areas, it’s like you are just working with the community, and you don’t grow. You just deal with the most common ailments….of course in the rural area you’ll never enter in a theatre like in the urban area” Pre-service nursing student

Others pointed out that some rural communities reject health workers and health care in general and prefer traditional medicine adding that such rural posts were not attractive to them.

“The real rural area is Nyadhuna... the patients have a poor attitude toward the nurses, and they prefer the TBAs [Traditional birth attendants]” Pre-service nursing student

“I think even before they post you, they should consider the community where they are taking you, because if I am taken to a community where I know I will be rejected, I cannot accept that one” Pre-service nursing student

Faith-based organizations (FBOs) which contribute approximately 40% of national healthcare mainly in rural and underserved areas were thought to pay the lowest salaries. This particular sentiment was shared amongst upgrading students who also felt that workloads in FBOs were often too high.

“when you go to some of the private hospitals especially the mission hospitals, I tell you that place is horrible because you see somebody with an experience of ten years in the same institution and cannot even afford a salary of KES 14,000 (USD 179.48) so what I mean is especially the mission hospitals, some of the mission hospitals the pay is very poor and the work load is too much…” Upgrading nursing student

Despite individual preferences both groups of trainees were of the opinion that all cadres of nurses (including ECNs and RNs) are adequately trained to work in any health facility, and that they should therefore be ready to work in lower level facilities in rural areas.

“what I think, like now there should be a channel of everybody having an opportunity to pass through the rural and the hospital because we are trained for all this” Upgrading nursing student

### Strategies to recruit and retain nurses in rural areas

Varied perceptions of strategies to recruit and retain nurses were reported in the SAQ (Table 2). Students generally felt compulsory rural service for government supported students was reasonable and that, for pre-service students, the greater responsibility in a rural area might be motivating. These positive work attributes could be enhanced by better housing and prospects for career advancement. Being able to choose the rural area to work in was also felt to be of some value while there was strong support for greater rural financial incentives as a means to attract people to such posts, with the majority of students suggesting that this should be up to 50% of basic salary. Pre-service students were significantly more likely than in-service students to agree with the statements on the motivating impact of greater responsibility and the importance of decent housing.

During FGDs, nursing students in both groups were mostly unaware of any interventions introduced in Kenya to recruit and retain health workers in rural areas. Some upgrading students were aware that rural hardship allowances ranging from KES 600 (USD 7.7) to KES 1300 (USD 16.6) per month are currently offered as a means to retain some cadres in remote areas, though they were felt to be insufficient to influence decisions on whether to work in the more remote provinces.

“… in government … sometimes you are in a … place … so remote, and you don’t get risk allowance … those who get hardship allowance get a maximum of KES 1300 (US$16.6) and something, that KES 1000 (USD 12.8) can it allow me to work in North Eastern? No.” Upgrading nursing student

Students also felt that if advertisements for public sector nursing jobs specified job location and facility type, potential applicants would be able to make more informed decisions when applying for posts. Students reported that such details are not provided for standard government posts, but have been included in advertisements for the recent short-term government contracts. Recruitment procedures for the private sector and FBOs were reported to be clearer than in the public sector, allowing potential applicants to know what role they are applying for and where they will be posted.

“Most of the NGOs’ adverts are more elaborate because they have job descriptions, qualifications and all that. And you realize that hospitals [Public] give you very little information about the job you intend to apply for, maybe they just say a KRHN nurse with a diploma and a working experience of this [number of] years but I find NGOs and CBOs (community-based organizations) more elaborate on what they want when they advertise their jobs.” Pre-service nursing student

In FGDs upgrading students supported the view that rural postings in health centers and dispensaries offered nurses a valuable opportunity to carry out managerial duties as in-charges (managers) of the facilities.

“… in the villages you will be performing your managerial functions, you will manage yourself, the drugs…” Upgrading nursing student

It was suggested that rural recruitment could be boosted by recruiting MTC students from rural areas, and perhaps training them in MTCs located in relatively remote areas. Many students favored the idea, and one upgrader argued that rural MTCs should implement a ‘quota system’ and restrict three quarters of their students to be recruited locally. However, others thought this strategy would be unlikely to have a major impact on rural recruitment in the face of persistent infrastructural problems. Moreover, high illiteracy levels in some marginalized communities where very few people attain the qualifications necessary for college education were cited as a major challenge meaning that the most marginalized would be unlikely to benefit fully.

Although likert scale responses indicated that students felt support from colleagues and supervisors was adequate in rural areas, during FGDs students argued that supervision was often focused on fault-finding, and that more supportive regular supervision would encourage nurses to practice in rural areas since they would feel less neglected.

“…I have been working in a rural set up where they do not come, but the moment they will hear that facility has a problem, they will come every month hunting you, stopping your salary, yeah…that is their idea. They do not give you support…supportive supervision” Upgrading nursing student

In the SAQ, about half the upgrading and pre-service students (49.4% and 52.5% respectively) felt that “a safe job with no risk of closing down or unemployment” was the most important factor when looking for a job, ranking this more highly than “a good income so that you do not have any worries about money”, “doing an important job that gives you a feeling of accomplishment”, or “working with people you like”. This was reflected during FGDs, where the new short-term contracts for specific rural posts were generally unpopular, with permanent public contracts preferred by both pre-service and upgrading trainees. Those disliking short contracts feared the lack of pension plan and long-term job security.

“Short term contracts, you can get a lot of money at a go but going to get another job is difficult, that money you may stay with it at home and spend it completely, what will you do?” Upgrading nursing student

In addition, delayed salary payments experienced by short-term hires came out as an issue of contention.

“…they just pay for one month then they carry forward the rest of the months, you see when a person is permanently employed at the end of each month at least…[they get their salary]” Upgrading nursing student

## Discussion

Inadequate human resources have been identified as a key constraint to scale up of health interventions [7,8]. Mal-distribution of health workers continues to be a global challenge with high concentration of health workers in urban areas while rural areas are understaffed [22]. This situation threatens equitable delivery of health services to people living in rural areas who are often less educated, poorer and with a higher disease burden. There has been a general neglect of health policies which focus on strengthening human resources for health, reflected in poor budget allocations and limited time within the national health agenda devoted to this topic in both developed and developing countries [5,20]. This paper provides evidence from Kenya about the challenges of recruiting and retaining nurses in rural posts, and the potential of a range of strategies to address this.

We studied responses from both pre-service and upgrading students. The latter were older, more likely to be married, to have children, and to have been born in a rural area than pre-service trainees. More upgraders reported that nursing was their first career choice. This may have reflected a survivor bias as upgraders interviewed had already worked in the profession for several years, or the declining popularity of the nursing over time, reflecting the reduced attractiveness of the profession (e.g. due to relative pay, prestige and support), or the wider range of career options available in more recent years. However, responses from the two groups in the SAQ and FGDs were broadly similar; the only significant differences in likert scale responses were that pre-service students were more positive about the potential impact of greater responsibility and improved housing on rural recruitment. Multivariable analysis showed that when other factors were controlled for, being an upgrading student had a significantly negative impact on perceptions of rural posts. The reasons for this are unclear, but could reflect their greater experience of the realities of rural posts, or a preference for urban areas making it more likely that an ECN will choose to upgrade. Kenya is planning to phase out ECNs, an approach already adopted in other African countries [23]. There is concern that as a result Kenya will have a more qualified nursing workforce which is more marketable locally and internationally, and less willing to work in rural areas. However, the data do not allow a direct comparison of RN and ECN preferences as all respondents were close to becoming RN graduates at the time of data collection.

Across both groups likert scale responses indicated generally negative perceptions of nursing posts in rural areas, although stated preferences were rarely strong. During FGDs trainees raised both positive and negative aspects of rural life. On the positive side they mentioned lower costs of living and more autonomy at work. On the negative side they cited poor infrastructure, inadequate education facilities and opportunities, higher workloads, and inadequate supplies and supervision, factors also highlighted in other studies [22]. Parents working in rural areas may also be forced to separate from their children who attend schools in urban areas, an issue also highlighted in South Africa[24]. Particular concern was expressed about working in communities dominated by other tribes, reflecting Kenya’s recent election-related violence.

Quantitative analysis showed that attitudes to living in rural areas were significantly positively affected by studying at the MTC furthest from Nairobi, and attitudes to working in rural areas were positively affected by being older. One might expect students born in a rural area to have more positive attitudes towards living and working in such locations. For example a review related to doctors found strong evidence that students with a rural origin are more likely to practice in a rural setting [20]. However, in this study, rural birth did not have a significant impact on attitudes once other factors where controlled for. However, such conclusions should be considered tentative given the relatively low Cronbach’s alpha associated with the PCA derived constructs and the limited explanatory power of the regression models.

Students expressed mixed views on the relative merits of working in private and public sectors. While pre-service students perceived commercial and NGO facilities to pay the highest salaries, upgraders emphasised that the FBO organisations had the lowest salaries despite their high workload. The fact that the FBO segment of the private sector is located predominately in rural areas, while the commercial segment is predominately in urban areas, may be a further factor influencing rural/urban job choices.

Quantitative and qualitative data indicated that students believed a number of strategies could work to improve rural recruitment and retention, with particular emphasis on the introduction of substantial rural allowances. Many studies point to the value of financial incentives to enhance rural recruitment and retention [25], although it has been argued that multidimensional programmes are more successful than those relying on financial incentives alone [20]. The implementation of such strategies requires clear definitions of rural areas to ensure equity and fairness in distribution of allowances [26], and the allocation of adequate funds to ensure sustainability [27].

Pre-service trainees perceived that increased responsibility would motivate nurses, a factor also noted in other studies [22]. FGD data also indicated that trainees thought more supportive supervision would encourage nurses to practice in rural areas. Other studies have highlighted the important role that managers can play in job satisfaction [23,28].While there is some evidence that improved infrastructure and supportive supervision improve retention [29,30], it is worth noting that infrastructural problems themselves limit the amount and frequency of supervisory visits to remote areas. Views of compulsory rural service were positive but not strongly supportive; other studies have found that the evidence that this has a positive long-term impact on rural-urban mismatch is weak [20].

Qualitative work revealed, importantly, that in a context of political instability, interventions aimed at improving recruitment and retention in rural areas may fail to meet their objectives. As a consequence of recent post election violence, trainees expressed concern about accepting rural posts where ethnic tensions may affect their personal security, highlighting that for rural recruitment strategies to be attractive to nurses, their basic safety needs must be assured [24]. The ability to select the location of a rural post was also highlighted as likely to encourage application during FGDs and reflected in likert scale results. Interestingly, the recently introduced short term, donor funded contracts that target critical human resource gaps in underserved areas were regarded as less attractive than standard government posts due to their lack of pension plans and job security, despite a stated policy that such posts be absorbed by the government at the end of the contract. However, applications for such posts are plentiful (Chief Nursing Officer, personal communication), probably reflecting both the strong supply of nursing graduates and the lack of alternative opportunities in Kenya.

A number of limitations should be highlighted. First for logistical reasons it was not possible to select a nationally representative sample of trainees. In addition, data were collected within a year of the post election violence which may have modified nurse’s perceptions of rural postings. Thirdly, the term “rural” is difficult to define [20] and its interpretation is likely to have varied across interviewees. For example, some may have considered district towns as urban, while others may have classified them as rural. However, the characteristics students identified for rural areas during FGDs were similar to those that we proposed during quantitative data collection. Other terms used in the SAQ may also have been open to varied interpretations such as “lifestyle”, “motivation” “stressful” and “decent housing”. The list of potential interventions assessed was not comprehensive; others identified as potentially useful in other studies include conducting training placements in rural settings, providing scholarships with an enforceable rural service agreement, accelerated promotion, and increased recognition by the employer or community [20,22,27].

Finally, the findings are based on the stated opinions of trainees and may not translate into actual career decisions. It is notable that while many strategies to improve recruitment and retention have been proposed in the literature [31], few studies have investigated their effectiveness. Further research is therefore necessary to investigate the extent to which the intentions of qualifying nurse graduates translate into career moves, and the effectiveness of interventions to influence recruitment and retention patterns over time. In developed countries, retrospective studies have been conducted to document the career paths of health workers [32] but similar databases are not available in Kenya, while results from the developed world are unlikely to be applicable to developing countries which have very different health labor markets and health workforce distribution [33].

## Conclusions

The issue of workforce shortage and mal-distribution is complex and not unique to the nursing cadre or to Kenya. Poor infrastructure, limited training opportunities, high workloads, inadequate supplies and supervision, undisclosed job locations for public sector jobs, and most recently political instability all continue to be barriers to successful rural recruitment and retention. Interestingly we found no suggestion that those born in or with experience working in rural areas are more willing to seek rural employment. While donor funded short-term contracts have increased recruitment in recent years, it is possible that their impact will be compromised by their unpopularity among nurses due to their lack of pension plans and job security. The most popular proposed policy intervention among respondents was the provision of additional financial incentives for rural posting, though these may be more effective if implemented as part of a multi-dimensional package. Such a package would require collaboration between economic and health policy-makers to earmark funding to not only secure salaries but also improve working conditions. It should also be accompanied by investment in information systems capable of monitoring its impact with rigor.

## Competing interests

The authors declare that they have no competing interests.

## Authors' contributions

ME and KM designed the study with contributions from CG, JW, ML and DB. DB, SM and ML performed quantitative analysis while KM and JW performed qualitative analysis of the data. All authors participated in the interpretation of the results. KM and SM drafted the first version of the manuscript, and all authors contributed to subsequent versions and revised it critically. All authors read and approved the final manuscript.

## Supplementary Material

Additional file 1 Characteristics of Respondents Click here for file

Additional file 2 Mean Likert scale scores for attitudes towards rural areas and policy interventions (minimum score 1, maximum 6) Click here for file

Additional file 3 PCA results showing loading of each statement on the first component for (a) Attitudes towards lifestyle in rural areas; and (b) Attitudes towards working in rural areas Click here for file

Additional file 4 Multivariable Regression results Click here for file
