# Supplementary material for: Attracting and retaining health workers in rural areas: investigating nurses’ views on rural posts and policy interventions
Source: BMC Health Serv Res. 2010 Jul 2;10(Suppl 1):S1. doi: 10.1186/1472-6963-10-S1-S1 (PMC2895745; doi:10.1186/1472-6963-10-S1-S1)
Supplement: Additional file 1 — Characteristics of Respondents [file 1472-6963-10-S1-S1-S1.docx]

## Table 1 - Characteristics of Respondents [Additional file 1]

|  | Pre-service (n=166) | Upgrading (n=179) | Total (n=345) |
| --- | --- | --- | --- |
| MTC (%): Nairobi | 55 (33.1%) | 64 (35.8%) | 119 (34.5%) |
| Murang’a | 48 (28.9%) | 31 (17.3%) | 79 (22.9%) |
| Meru | 29 (17.5%) | 29 (16.2%) | 58 (16.8%) |
| Kakamega | 34 (20.5%) | 55 (30.7%) | 89 (25.8%) |
| Sex: female (%) | 113 (68.1) | 147 (82.1) | 260 (75.4) |
| Mean age in years (sd) | 23.96 (2.3) | 37.53 (6.4) | 31 (8.4) |
| Marital status: married (%) | 18 (10.8) | 138 (77.1) | 156 (45.2) |
| Any children (%) | 25 (15.1) | 159 (88.8) | 184 (53.33) |
| Educated father*(%) | 129 (77.7) | 103 (57.5) | 232 (67.3) |
| Educated mother* (%) | 120 (72.3) | 72 (40.2) | 192 (55.7) |
| Born in a rural area (%) | 95 (57.2) | 133 (74.3) | 228 (66.1) |
| Scholarship recipient for current course (%) | 24 (14.5) | 10 (5.6) | 34 (9.9) |

*a parent was considered educated if they had at least completed primary education
