# Supplementary material for: Attracting and retaining health workers in rural areas: investigating nurses’ views on rural posts and policy interventions
Source: BMC Health Serv Res. 2010 Jul 2;10(Suppl 1):S1. doi: 10.1186/1472-6963-10-S1-S1 (PMC2895745; doi:10.1186/1472-6963-10-S1-S1)
Supplement: Additional file 2 — Mean Likert scale scores for attitudes towards rural areas and policy interventions (minimum score 1, maximum 6) [file 1472-6963-10-S1-S1-S2.docx]

## Table 2 - Mean Likert scale scores for attitudes towards rural areas and policy interventions (minimum score 1, maximum 6)

|  |  | Pre-service | Upgrading | Total |
| --- | --- | --- | --- | --- |
|  |  | mean (CI) | mean (CI) | mean (CI) |
| Perceptions of life and work in rural areas | | | | |
|  | Housing is good in rural areas | 2.8(2.6-3.1) | 3.2(2.9-3.5) | 3.0(2.8-3.2) |
|  | Quality of life in rural areas is very good | 3.4(3.2-3.7) | 3.4(3.1-3.7) | 3.4(3.2-3.6) |
|  | The lifestyle you have in rural areas appeals to me | 3.2(2.9-3.4) | 3.3(3.0-3.5) | 3.2(3.0-3.4) |
|  | The social life in rural areas in enjoyable | 3.3(3.0-3.6) | 3.7(3.5-4.0) | 3.5(3.3-3.7) |
|  | Being posted in a rural area would appeal to me | 3.7(3.5-3.9) | 3.3(3.1-3.5) | 3.5(3.3-3.7) |
|  | I would feel scared if I had to work in a rural area | 2.0(1.8-2.2) | 2.5(2.2-2.7) | 2.2(2.1-2.4) |
|  | Bringing up children in rural areas is difficult | 2.5(2.2-2.7) | 2.7(2.5-3.0) | 2.6(2.4-2.8) |
|  | Working in rural areas means you are without support from colleagues/supervisors | 1.8(1.6-2.0) | 1.7(1.5-1.9) | 1.7(1.6-1.9) |
|  | You can earn more money when you work in a rural area | 3.3(3.0-3.6) | 2.7(2.4-3.0) | 3.0(2.8-3.2) |
|  | You can obtain advancement in your career quickly if you choose a rural position | 2.9(2.7-3.2) | 2.6(2.3-2.8) | 2.8(2.6-2.9) |
|  | Working in rural areas is not stressful at all | 2.7(2.4-2.9) | 2.9(2.6-3.1) | 2.8(2.6-3.0) |
|  | Living in a city is stressful | 3.7 (3.4-3.9) | 3.9 (3.7-4.2) | 3.8 (3.6-4.0) |
| Perceptions of strategies to recruit and retain nurses in rural areas | | | | |
|  | Making it compulsory for graduates from Gok funded colleges to spend time in a rural health facility is a good thing | 3.7(3.4-4.0) | 3.9(3.6-4.2) | 3.8(3.6-4.0) |
|  | Paying more to nurses who work in disadvantaged or very remote areas is fine | 5.6(5.4-5.8) | 5.7(5.5-5.8) | 5.6(5.5-5.7) |
|  | Giving more responsibilities to nurses is a good way to motivate them | 4.4(4.1-4.7) | 3.5(3.2-3.8) | 3.9(3.7-4.1) |
|  | If I have work in a rural area it is important to me to be able to chose which rural area | 4.5(4.2-4.8) | 4.7(4.5-5.0) | 4.6(4.5-4.8) |
|  | If decent housing was provided with posts in rural areas I would be happy to go | 4.5(4.3-4.8) | 3.9(3.6-4.1) | 4.2(4.0-4.4) |
|  | For your career advancement, each year spent as a nurse in a remote or disadvantaged area should count twice as much as anywhere else | 4.5(4.3-4.8) | 4.2(4.0-4.5) | 4.4(4.2-4.6) |

## All the questions were rated on a scale from 1= strongly disagree 2= moderately disagree 3= somewhat disagree 4= somewhat agree 5= moderately agree to 6= strongly agree
