# Supplementary material for: Attracting and retaining health workers in rural areas: investigating nurses’ views on rural posts and policy interventions
Source: BMC Health Serv Res. 2010 Jul 2;10(Suppl 1):S1. doi: 10.1186/1472-6963-10-S1-S1 (PMC2895745; doi:10.1186/1472-6963-10-S1-S1)
Supplement: Additional file 3 — PCA results showing loading of each statement on the first component for (a) Attitudes towards lifestyle in rural areas; and (b) Attitudes towards working in rural areas [file 1472-6963-10-S1-S1-S3.docx]

## Table 3 - PCA results showing loading of each statement on the first component for (a) Attitudes towards lifestyle in rural areas; and (b) Attitudes towards working in rural areas

|  |  | Loading of each statement on the first component |
| --- | --- | --- |
| (a) Attitudes towards lifestyle in rural areas | | |
| 1. | Housing is good in rural areas | 0.415 |
| 2. | Quality of life in rural areas is very good | 0.557 |
| 3. | The lifestyle you have in rural areas appeals to me | 0.526 |
| 4. | The social life in rural areas is enjoyable | 0.49 |
| (b) Attitudes towards working in rural areas | | |
| 5. | Working in rural areas means you are without support from colleagues/supervisors | 0.294 |
| 6. | You can earn more money when you work in a rural area | 0.37 |
| 7. | You can obtain advancement in your career quickly if you choose a rural position | 0.384 |
| 8. | Working in rural areas is not stressful at all | 0.266 |
| 9. | Being posted in a rural area would appeal to me | 0.462 |
| 10. | I would feel scared if I had to work in a rural area | 0.477 |
| 11. | Bringing up children in rural areas is difficult | 0.344 |
