# Supplementary material for: Attracting and retaining health workers in rural areas: investigating nurses’ views on rural posts and policy interventions
Source: BMC Health Serv Res. 2010 Jul 2;10(Suppl 1):S1. doi: 10.1186/1472-6963-10-S1-S1 (PMC2895745; doi:10.1186/1472-6963-10-S1-S1)
Supplement: Additional file 4 — Multivariable Regression results [file 1472-6963-10-S1-S1-S4.docx]

## Table 4 - Multivariable Regression results

| Independent Variables | Regression Coefficients | |
| --- | --- | --- |
|  | Attitudes towards lifestyle in rural areasOverall p value = <0.001R squared = 0.0872n= 343 | Attitude towards working in rural areasOverall p value = 0.001R squared = 0.0779n= 340 |
| Upgrading student | -0.32 | -1.03*** |
| Age (yrs) | 0.01 | 0.03* |
| Male | 0.14 | 0.21 |
| Single | 0.27 | 0.02 |
| Any children | 0.65 | 0.23 |
| Born in a rural area | 0.07 | 0.25 |
| Meru MTC^1^ | -0.09 | -0.18 |
| Kakamega MTC^1^ | 0.52** | 0.28 |
| Murang’a MTC^1^ | -0.40 | -0.07 |
| Constant | -0.79 | -0.67 |

## *p=0.05 *** p<0.001

## ^1^ Compared to Nairobi MTC as the base case
